# *Staphylococcus pettenkoferi*: an underrecognized species with clinically relevant antimicrobial resistance. A comprehensive genomic European study

**DOI:** 10.1080/22221751.2026.2724635

**Published:** 2026-09-16

**Authors:** Chloé Magnan, Matthieu Curtil-Dit-Galin, Madjid Morsli, Vincent Cattoir, Caroline Piau-Couapel, Bo Söderquist, Barbara C. Kahl, Olivier Denis, Kenza Rwayane, Stéphane Corvec, Patrice François, Florian Salipante, Alix Pantel, Catherine Dunyach-Remy, Frédéric Laurent, Jean-Philippe Lavigne, on behalf of the European Society of Clinical Microbiology and Infectious Diseases (ESCMID) Study Group for Staphylococci and Staphylococcal Diseases (ESGS)

**Affiliations:** a Department of Microbiology and Hospital Hygiene, VBIC, INSERM U1047, Univ Montpellier, CHU Nîmes, Nîmes, France; b CIRI, Inserm U1111, Université Claude Bernard Lyon 1, CNRS UMR5308, ENS Lyon, Lyon, France; c Department of Clinical Microbiology, CHU Rennes University Hospital, Rennes, France; d Department of Laboratory Medicine, Faculty of Medicine and Health, Örebro University, Örebro, Sweden; e Institute of Medical Microbiology, University Hospital Münster, Münster, Germany; f Department of Microbiology, Hopital Universitaire de Bruxelles, ULB, Bruxelles, Belgium; g Service de Bactériologie et des Contrôles Microbiologiques, INSERM, CNRS, Immunology and New Concepts in ImmunoTherapy, INCIT, UMR 1302/EMR6001, Nantes Université, Univ Angers, CHU Nantes, Nantes, France; h Genomic Research Laboratory, Service of Infectious Diseases, Geneva University Hospitals and Faculty of Medicine, Geneva, Switzerland

**Keywords:** Coagulase-negative staphylococci, methicillin resistance, molecular epidemiology, phylogenomics, whole genome sequencing

## Abstract

*Staphylococcus pettenkoferi* has increasingly been reported as a human pathogen, with virulence potential recently confirmed *in vivo*. However, its genomic diversity and antimicrobial resistance remain insufficiently characterized. This study, conducted within the PETTENK-OS project of the ESCMID Study Group for Staphylococci and Staphylococcal Diseases (ESGS), aimed to characterize the genomic diversity and resistome of this underrecognized coagulase-negative staphylococcus (CoNS). Whole-genome sequencing was performed on 61 European clinical isolates (mainly bloodstream infections and osteomyelitis) from 17 hospitals in five countries, plus two reference strains. Antimicrobial susceptibility profiles were determined by disk diffusion and broth microdilution (2025 EUCAST breakpoints). Phylogenetic analyses clustered the 63 isolates into two major clades: Clade I (n = 51; subclades I-A, n = 47, and I-B, n = 4) and Clade II (subclades II-A, n = 8, and II-B, n = 3). Susceptibility rates were below 70% for cefoxitin, erythromycin, and levofloxacin, whereas high susceptibility (>90%) was observed for vancomycin, teicoplanin, dalbavancin, aminoglycosides, oxazolidinones, trimethoprim-sulfamethoxazole, fusidic acid, and tetracycline. Nearly all isolates (92.1%) were fosfomycin-resistant. Daptomycin MIC50/90 values (1/2 mg/L) were elevated, including eight resistant isolates carrying mutations in *walKR*, *cls1*, and/or *cls2* genes. Twenty-two isolates (35.4%) were multidrug-resistant, and 18 (28.6%) were methicillin-resistant, all in subclade I-A and mecA-positive within SCC*mec* type IV. This study provides a comprehensive genomic and phenotypic assessment of antimicrobial resistance in *S. pettenkoferi*, revealing widespread multidrug-resistant lineages, including ceftobiprole- and daptomycin-resistant isolates across distinct clades. These findings underscore the need for continued surveillance and improved diagnostics for this increasingly encountered CoNS species.

## Introduction

Coagulase-negative staphylococci (CoNS) have gained increasing recognition for their involvement in a wide spectrum of infections, extending far beyond their traditional classification as harmless mucocutaneous commensals [1]. Historically overshadowed by the more virulent coagulase-positive *Staphylococcus aureus* [2], CoNS are now established as important opportunistic pathogens. Their ability to form biofilms, particularly on indwelling medical devices such as intravascular catheters, prosthetic heart valves, and orthopaedic implants, combined with the acquisition of intrinsic and adaptative antimicrobial resistance mechanisms, poses significant challenges for clinical management [3,4]. Additionally, CoNS are capable of evading host immune defences [5,6], allowing them to persist and contribute to chronic infections in vulnerable populations. Consequently, accurate species-level identification has become essential to support the clinical interpretation of CoNS isolates and, together with microbiological and clinical findings, to distinguish true pathogens from contaminants. In recent years, several CoNS species have been implicated in healthcare-associated infections, including documented bacteraemia outbreaks caused by *Staphylococcus capitis* [7] and *Staphylococcus haemolyticus* [8]. Other species, notably *Staphylococcus epidermidis* [9] and *Staphylococcus lugdunensis* [10], have emerged as important aetiological agents of bone and joint infections (BJIs), prosthetic joint infections (PJIs) [11], and diabetic foot infections (DFIs) [12]. Advances in diagnostic technologies such as MALDI-TOF mass spectrometry have markedly improved the detection and differentiation of CoNS species [1], revealing a level of pathogenic diversity previously underestimated.

*Staphylococcus pettenkoferi*, a CoNS species first identified in 2002 [13], has increasingly been associated with severe human infections, including bacteraemia and bone and joint infections, particularly in patients with underlying comorbidities [14–16]. Its pathogenic potential is supported by its ability to survive in human blood, keratinocytes, and macrophages as well as its capacity to form biofilms, and to harbour virulence-associated determinants linked to persistence and pathogenicity [17]. Recent *in vivo* studies using a zebrafish embryo model further demonstrated strain-dependent virulence following bath infection of wounded larvae, suggesting a possible role in complex infections such as diabetic foot osteomyelitis (DFOM) and bacteraemia [18]. Despite growing recognition of *S. pettenkoferi* as an underrecognized pathogen in severe DFIs [19–22], knowledge of its antimicrobial resistance patterns remains limited, and no standardized susceptibility profiles have yet been proposed by international committees.

To address this gap, we conducted a large-scale investigation within the PETTENK-OS project led by the European Society of Clinical Microbiology and Infectious Diseases (ESCMID) Study Group for Staphylococci and Staphylococcal Diseases (ESGS). We analyzed 61 clinical isolates collected across 17 European hospitals from five countries, predominantly from blood cultures and BJI cases, with or without associated DFIs/DFOMs. This study represents the first comprehensive assessment of *S. pettenkoferi* integrating phylogenetic characterization, phenotypic antimicrobial susceptibility testing, and in-depth resistome analysis.

## Materials and methods

### Bacterial strain collection

This multicenter study included 61 *S. pettenkoferi* clinical isolates collected from 17 hospitals across five European countries between 2012 and 2024. Only one isolate per patient and per clinical episode was included in the study; duplicate isolates and longitudinal isolates from the same patient were excluded. In France, isolates were obtained from Rennes University Hospital (*n* = 12), Nîmes University Hospital (*n* = 9), and Nantes University Hospital (*n* = 4). The French National Reference Center (NRC) for Staphylococci in Lyon additionally provided nine isolates originating from nine different French hospitals: Albi Hospital, Bicêtre Hospital (AP-HP), Lille University Hospital, Armentières Hospital, Rodez Hospital, Bordeaux University Hospital, Tourcoing Hospital, Poitiers University Hospital, and the Hospices Civils de Lyon. In Sweden, 11 isolates were collected, including 10 from Örebro University Hospital and one from Växjö Hospital. Ten isolates were obtained from Münster University Hospital (Germany), five from CHU UCL Namur (Belgium), and one from Geneva University Hospitals (Switzerland).

Among the 61 isolates, 35 (57.4%) were recovered from blood cultures, 19 (31.1%) from BJIs, 3 (4.9%) from skin colonization, 2 (3.3%) from biological fluids (one ascitic fluid and one preservation solution), 1 (1.6%) from tissue biopsy, and 1 (1.6%) from pus. Of all bone and tissue samples, 15 (24.6%) were associated with DFIs. Two previously described *S. pettenkoferi* strains were included for comparative genomic analyses: the type strain CCUG51270^T^ and the reference strain K6999, both originally recovered from blood cultures in Germany (Munich and Münster, respectively). The complete collection is presented in Supplementary Table 1. Species identification was performed using MALDI-TOF mass spectrometry (MALDI Biotyper®, Bruker, Billerica, MA, USA). All isolates were stored at -20°C in cryotubes until further processing.

### Antimicrobial susceptibility testing

Antimicrobial susceptibility testing was performed using both disk diffusion and broth microdilution methods. Each isolate was subcultured twice on trypticase soy agar supplemented with 5% sheep blood (bioMérieux, Marcy l'Étoile, France), and incubated at 37°C for 48 h due to the slow growth of the species. A 0.5 McFarland suspension in sterile 0.9% NaCl solution was prepared for testing.

Disc diffusion was performed on Mueller-Hinton agar using antibiotic discs (Bio-Rad, Marnes-la-Coquette, France) according to the 2025 EUCAST breakpoint guidelines (version 15.0) (https://www.eucast.org/). The following antibiotics were tested: penicillin G, ampicillin, cefoxitin, erythromycin, clindamycin, quinupristin-dalfopristin, kanamycin, tobramycin, gentamicin, tetracycline, norfloxacin, fusidic acid, rifampicin, trimethoprim-sulfamethoxazole, and fosfomycin. Inhibition zone diameters (IZDs) were measured using SIRscan Micro® software (i2a, Montpellier, France). For erythromycin-resistant and clindamycin-susceptible isolates, the D-zone test was performed according to EUCAST recommendations to differentiate inducible MLS_B_ from efflux-mediated resistance. Isolates resistant to both erythromycin and clindamycin were classified as exhibiting a constitutive MLS_B_ phenotype. Minimum inhibitory concentrations (MICs) of ampicillin, ceftaroline, ceftobiprole, clindamycin, dalbavancin, delafloxacin, levofloxacin, linezolid, rifampicin, tedizolid, vancomycin, teicoplanin, daptomycin, and trimethoprim-sulfamethoxazole were determined using Sensititre® microdilution plates (FR2STENT, Thermo Fisher Scientific™, Waltham, MA, USA), following the manufacturer's instructions. Resistant MIC results for ceftaroline, ceftobiprole, and dalbavancin were systematically confirmed using gradient strip tests (Liofilchem® MTS, Roseto degli Abruzzi, Italy). In addition, isolates found resistant to levofloxacin and/or delafloxacin by broth microdilution despite susceptibility to norfloxacin by disk diffusion, in accordance with EUCAST recommendations for screening fluoroquinolone resistance, were re-evaluated by gradient strip testing (bioMérieux for levofloxacin; Liofilchem® MTS for delafloxacin). Rifampicin MICs were re-evaluated by gradient strip testing (bioMérieux) whenever discrepant results were observed between disk diffusion and broth microdilution. Broth microdilution (UMIC®, Bruker, Champs-sur-Marne, France) was used to confirm vancomycin, teicoplanin, and daptomycin MICs. Interpretation followed the EUCAST breakpoints for *Staphylococcus* spp. (version 15.0), applying *S. aureus* or CoNS breakpoints when species-specific thresholds were unavailable [23]. For ceftaroline, the *S. aureus* breakpoint for non-pneumonia indications was used, and for delafloxacin, the *S. aureus* breakpoint for skin and skin-structure infections was applied. Older breakpoints were used for fosfomycin (CA-SFM 2020 v1.2) and kanamycin (EUCAST v13.1) as no updated diffusion cut-offs exist [24,25]. IZD and MICs were independently interpreted by two operators, with a third operator resolving discrepancies.

### DNA extraction and whole-genome sequencing

All isolates including the two reference strains, were cultured aerobically for 48 h at 37°C on Columbia blood agar (5% sheep blood; bioMérieux). Genomic DNA was extracted using the DNeasy UltraClean Microbial Kit (Qiagen, Courtaboeuf, France) with an added enzymatic lysis step (10 µL lysostaphin at 1 mg/mL per sample). DNA concentration was quantified using a Qubit® fluorometer (Thermo Fisher Scientific), and extracts with ≥50 ng/µL were used for sequencing. Illumina paired-end libraries were prepared from 250 ng of DNA using the DNA Prep Kit (Illumina, San Diego, CA, USA). Library quality was assessed using the TapeStation system and High Sensitivity D1000 ScreenTape assay kit (Agilent, Sainte Claire, MI, USA). Whole-genome sequencing (WGS) was performed on an Illumina MiSeq platform using a 39 h run, generating 2 × 250-bp paired-end reads. For the type strain CCUG51270^T^ and SP165, long-read sequencing was performed to obtain a complete genome assembly. Libraries were prepared from 200 ng of DNA using the Rapid Barcoding Kit 24 V14 (Oxford Nanopore Technologies™, Oxford, UK), purified with AMPure XP beads, eluted in 15 µL, and sequencing adapters were added according to the manufacturer’s instructions. The final library was loaded onto an R10.4.1 flow cell and sequenced for 24 h on a MinION device (Oxford Nanopore Technologies™).

### *In silico* analysis

Data quality was assessed using FastQC-Read Quality reports QC (version 0.12.1). De novo assembly was performed with SPAdes software (version 3.13.1). The complete hybrid (Illumina and Oxford Nanopore) genome assembly of the type strain *S. pettenkoferi* CCUG51270^T^ was used as the reference genome for species confirmation and comparative genomic analyses. Species confirmation used the Type-Strain Genome Server (https://tygs.dsmz.de/user_requests/new) and the Orthologous Average Nucleotide Identity Tool (OAT) (version 0.93.10). Identification was confirmed using thresholds of ≥70% for digital DNA-DNA hybridization (dDDH) and ≥95% for average nucleotide identity (ANI) relative to the type strain *S. pettenkoferi* CCUG51270^T^. For the divergent isolate LSP1043, ANI and dDDH were additionally determined relative to the type strains of the three closest non-*S. pettenkoferi* species (*Staphylococcus argensis* CIP110904^T^, *Staphylococcus massiliensis* CCUG55927^T^, and *Staphylococcus marylandisciuri* CCUG76423^T^). Genomic assembly statistics and annotation features were calculated using the DDBJ Fast Annotation and Submission Tool (https://dfast.ddbj.nig.ac.jp/).

To place the study isolates in a broader phylogenetic context, publicly available *S. pettenkoferi* genome assemblies were retrieved from the NCBI Genome database. After exclusion of the 63 genomes generated in the present study, duplicate assemblies and low-quality genomes were removed. Remaining assemblies were filtered using the following quality criteria: fewer than 100 contigs >500 bp, N50 ≥ 50 kb, genome size between 2.4 and 2.6 Mb, and sequencing coverage ≥30×. Species assignment was confirmed using ANI (≥96%) and dDDH (≥70%) relative to the type strain CCUG51270^T^. The five public genomes fulfilling all inclusion criteria were retained for phylogenetic analysis (Supplementary Table 2).

Whole-genome alignment was generated with Parsnp (v1.2), using the complete hybrid assembly of the type strain *S. pettenkoferi* CCUG51270^T^ as the reference. The resulting core genome XMFA alignment was converted to multi-FASTA format using HarvestTools (v1.2). Pairwise SNP distances were calculated from the core-genome alignment using snp-dists (v0.8.2), generating a matrix of pairwise SNP distances based on variable sites shared across all genomes. Phylogenetic reconstruction used IQ-TREE (v2.3) with ModelFinder for automatic substitution model selection; branch support was assessed using 1,000 ultrafast bootstraps and 1,000 SH-aLRT replicates. Tree visualization and annotation were performed using the Interactive Tree Of Life (iTOL) online tool (https://itol.embl.de/). For pangenome analyses, genomes were annotated using Prokka (v1.14) and the pangenome was generated using Roary (v3.13.0) (95% BLASTp identity threshold; core genes defined as present in ≥99% of isolates). Core gene alignment was used to infer a phylogeny using the BioNJ algorithm under the Kimura 2-parameter (K2P) model with 1,000 bootstraps, as implemented in SeaView. Gene presence/absence was visualized using the Phandango online tool. Antimicrobial resistance genes were identified using ResFinder (v4.6.0; 80% nucleotide identity and 60% minimum length thresholds) and SCCmecFinder (v1.2; 90% nucleotide identity and 60% minimum length thresholds) available on the Center for Genomic Epidemiology platform (http://www.genomicepidemiology.org/services/). To validate SCCmecFinder subtype assignments, SCC*mec* regions from the representative SCC*mec* IVd(2B) isolate SP165 (complete hybrid genome assembly) and the SCC*mec* IVa(2B) isolate SPBel010 were extracted by BLAST. The extracted SCC*mec* regions, together with the partial SCC*mec* IVd(2B) reference sequence of *Staphylococcus aureus* JCSC4469 (GenBank accession AB097677.1), the complete SCC*mec* IVd(2B) element of *S. aureus* S145 [26] (BioSample SAMN24290962; doi:10.1016/j.jgar.2022.12.011), and the SCC*mec* IVa sequence of *S. aureus* JCSC1968 (GenBank accession AB063172), were independently annotated using Bakta (implemented in Proksee) and DFAST. Gene organization and synteny were subsequently visualized using the CAGECAT web server (clinker-based gene cluster comparison; cagecat.bioinformatics.nl). Mutations associated with antibiotic resistance were investigated using sequence alignment tools (https://www.ebi.ac.uk/jdispatcher/msa/clustalo).

### Statistical analysis

Statistical analyses were conducted using R software (version 4.3.2). Quantitative variables were expressed as medians with interquartile ranges (IQR), and compared using the Wilcoxon rank-sum test or Kruskal-Wallis test, as appropriate. Qualitative data were expressed as counts and percentages, and compared using Fisher’s exact test. A *p*-value < 0.05 was considered statistically significant. For comparisons of phenotypic and genotypic antimicrobial resistance across phylogenetic clades, *p*-values were additionally adjusted for multiple testing using the Benjamini-Hochberg false discovery rate (FDR) procedure, and the corresponding q-values were reported alongside the original *p*-values. A *p*-value < 0.05 was considered statistically significant for all analyses except comparisons of antimicrobial resistance profiles across phylogenetic clades, for which a q-value < 0.05 was considered statistically significant.

## Results

### Genome statistics and phylogenetic analysis

This multicenter study analyzed 61 *S. pettenkoferi* isolates collected between 2012 and 2024 from 17 European hospitals in five different countries. Most isolates originated from blood cultures (57.4%) and BJIs (31.1%), with additional sources including skin colonization, body fluids, tissue biopsies, and pus. WGS was performed to investigate genomic diversity and phylogenetic structure.

Species assignment was confirmed for all genomes by ANI scores ≥98% (Supplementary Table 3 and Supplementary Figure 1) and dDDH values >84% (Supplementary Table 3) relative to the type strain CCUG51270^T^, thereby confirming species-level identification. The median genome size was 2,461,288 bp, with a median G + C content of 38.9% (Table 1). Median counts included 2,341 coding sequences (CDS), 5 rRNA genes, and 59 tRNA genes, closely matching the characteristics of the type strain CCUG51270^T^ and the reference strain K6999. The median number of rRNA genes was five, although this value is likely underestimated because rRNA operons are repetitive regions that are frequently collapsed or incompletely assembled in short-read draft genomes. In contrast, the complete hybrid assembly of the type strain CCUG51270^T^ contained 19 rRNA genes. Detailed genome assembly and annotation statistics for each individual isolate are provided in Supplementary Table 4.

**Table 1.** Genomic characteristics of the 61 *S. pettenkoferi* clinical isolates, the type strain CCUG51270^T^, and the reference strain K6999.

Characteristic	All genomes (*n* = 61) Median [IQR]	CCUG51270^T^	K6999
Total Sequence Length (bp)	2,461,288 [2,439,537–2,478,015]	2,449,400	2,472,910
Number of Sequences	30 [27–34]	1	30
Longest Sequences (bp)	369,775 [333,514–542,127]	2,449,400	333,515
N50 (bp)	184,923 [139,942–224,376]	2,449,400	147,771
Gap Ratio (%)	0.0078 [0. 0040–0. 0121]	0.0000	0.0040
GCcontent (%)	38.9 [38.8–38.9]	39.1	38.9
Number of CDSs	2,341 [2,321–2,360.8]	2,317	2,346
Average Protein Length	291.4 [290.2–292.3]	291.6	292.9
Coding Ratio (%)	83.3 [83.1–83.3]	82.8	83.4
Number of rRNAs	5 [5–7]	19	6
Number of tRNAs	59 [57–60]	61	58
Number of CRISPRs	0	0	0

IQR = interquartile range. Values are reported as median [IQR] for the 61 collected isolates genomes and as individual values for strains CCUG51270^T^ and K6999.

**Figure 1.**
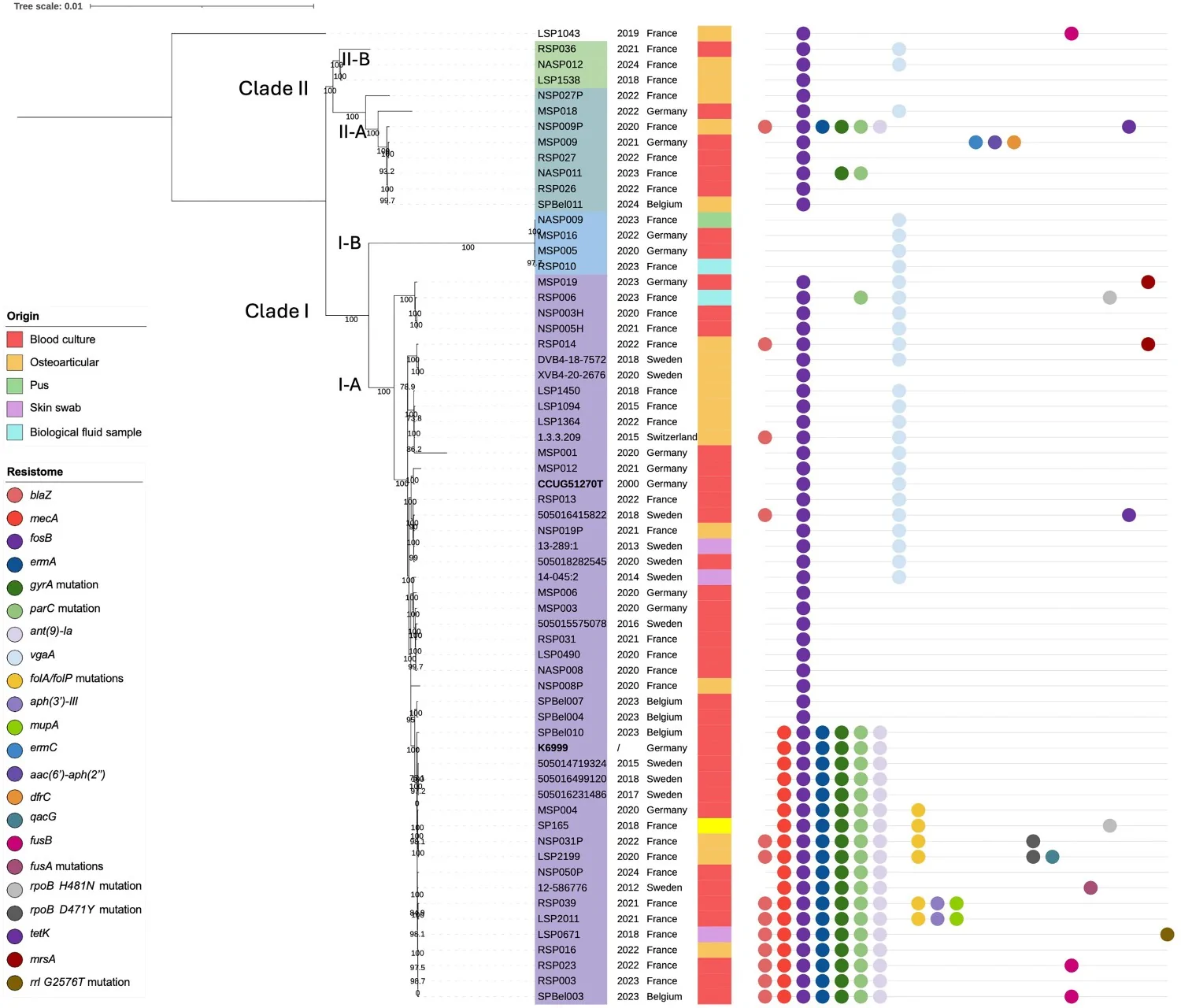
Unrooted whole-genome phylogeny of the 61 *S. pettenkoferi* clinical isolates, the type strain CCUG51270^T^, and the reference strain K6999. Maximum-likelihood tree inferred from core-genome alignment (IQ-TREE, ModelFinder; with branch support estimated from 1,000 ultrafast bootstrap and 1,000 SH-aLRT replicates). Visualisation performed using the iTOL online platform (https://itol.embl.de/). Clades (I, II) and subclades (I-A, I-B, II-A, II-B) are colour-coded. Outer rings display country, year of isolation, and clinical source.

A whole-genome alignment including all clinical isolates and the two reference strains revealed two major clades, designated Clade I (*n* = 51) and Clade II (*n* = 11) (Figure 1). Clade I was subdivided into subclades I-A (*n* = 47) and I-B (*n* = 4), whereas Clade II comprised subclades II-A (*n* = 8) and II-B (*n* = 3). One isolate (LSP1043) was markedly divergent and formed a divergent outgroup. Despite its divergent phylogenetic position, ANI and dDDH analyses confirmed its assignment to *S. pettenkoferi*. Comparisons with the type strains of the three closest non-*pettenkoferi* species (*S. argensis*, *S. massiliensis*, and *S. marylandisciuri*) yielded values well below the accepted species thresholds (Supplementary Table 3), excluding taxonomic misclassification. Pangenome-based clustering supported the same overall phylogenetic structure (Supplementary Figure 2). Clade I isolates were predominantly recovered from blood cultures (*n* = 36) and bone and soft tissue samples (*n* = 15), including 12 DFI-associated samples; the remaining isolates originated from other clinical sites (*n* = 8). Within this clade, subclade I-A (*n* = 47) encompassed the broadest clinical spectrum (29 blood cultures, 13 osteoarticular samples, three skin colonization samples, one ascitic fluid sample, and one tissue biopsy) and included both reference strains. Subclade I-B grouped four genomically similar isolates from distinct locations (two blood cultures, one pus sample, and one preservation solution). Clade II included eight isolates in subclade II-A (five blood cultures, three osteoarticular samples) and three French isolates in subclade II-B (two DFOM samples and one blood culture).

To assess whether this phylogenetic structure extended beyond the present European collection, five high-quality publicly available *S. pettenkoferi* genomes from the United States, Canada, and the United Kingdom, meeting the predefined inclusion criteria, were incorporated into the phylogenetic analysis (Supplementary Table 2). Their inclusion did not modify the overall tree topology or the definition of Clades I and II (Supplementary Figure 3). However, as these five genomes represent only a small fraction of the non-study assemblies publicly available for this species, this limited external dataset should be regarded as providing preliminary, rather than definitive, support for the clade structure identified in this study. High resolution SNP analysis identified several clusters of closely related isolates (Supplementary Table 5.A). The NSP003H/NSP005H (Nîmes, France) and XVB4-20-2676/DVB4-18-7572 (Örebro, Sweden) pairs were genetically identical or nearly identical (0 and 1 SNP difference, respectively). Three additional Swedish isolates (505016499120, 505014719324, and 505016231486) were closely related, with ≤60 SNPs differences. The four isolates (NASP009, RSP010, MSP005, and MSP016) from subclade I-B also displayed high genomic similarity despite originating from different cities and countries (Figure 1 and Supplementary Table 5.A). Two isolates from Nîmes and Lyon (NSP031P and LSP2199) differed by ≤53 SNPs. All methicillin-resistant *S. pettenkoferi* (MRSP) isolates within subclade I-A clustered tightly, with pairwise SNP distances of 53–401 (Supplementary Table 5.B).

Sampling years ranged from 2012 to 2024, with significant temporal differences across phylogenetic groups. Subclade I-A included a larger proportion of isolates collected before 2020 (*p* = 0.020). No significant associations were observed between clades and country of origin (*p* = 0.6), clinical sample type (*p* = 0.2), or DFI-related sampling (*p* = 0.11).

### Antimicrobial resistance profiles of *S. pettenkoferi* isolates

Antimicrobial susceptibility profiles of all 63 isolates (61 clinical isolates and two reference strains), including IZD_50/90_ and MIC_50/90_ values, are presented in Table 2. Using *S. aureus* and *S. saprophyticus* EUCAST breakpoints, penicillin G and ampicillin susceptibility rates were 63.5% (*n* = 40) and 69.8% (*n* = 44), respectively. However, these cut-offs are not recommended for CoNS and may misclassify susceptibility in *S. pettenkoferi.* Cefoxitin screening identified 43 isolates (68.3%) as methicillin-susceptible. For fifth-generation cephalosporins, 57 isolates (90.5%) were susceptible to ceftaroline and 48 (76.2%) to ceftobiprole, using *S. aureus* breakpoints. MIC_50/90_ values were ≤0.12/1 mg/L for ceftaroline and 1/8 mg/L for ceftobiprole. Notably, all isolates classified as “susceptible at increased exposure” for ceftaroline (*n* = 6), as well as all ceftobiprole-resistant isolates (*n* = 15), belonged to the cefoxitin-resistant group (*n* = 20).

**Table 2.** Antimicrobial susceptibility profiles of the 61 *S. pettenkoferi* clinical isolates, the type strain CCUG51270^T^, and the reference strain K6999.

Diffusion assay	Range (mm)	S ≥ cutoff (mm)	IZD_50_ (mm)	IZD_90_ (mm)	Susceptibility (%)
Penicillin G	6–40	26*	32	6	63.5
Ampicillin	6–40	18**	31	6	69.8
Cefoxitin	6–31	22	23	6	68.3
Erythromycin	6–42	21	35	6	65.1
Clindamycin	6–41	22	35	6	76.2
Quinupristin-Dalfopristin	26–47	21	35	31	100
Norfloxacin	6–43	17	36	6	73.0
Rifampicin	10–54	30	40	35	93.7
Trimethoprim-sulfamethoxazole	8–40	14–17	32	22	93.7
Kanamycin	6–40	22***	32	28	95.2
Tobramycin	15–45	20	36	32	98.4
Gentamicin	12–46	22	36	32	98.4
Fusidic acid	6–57	24	40	35	92.1
Fosfomycin	6–22	23****	6	6	7.9
Tetracycline	10–43	22	40	35	96.8

Micro-dilution assay (Sensititre^TM^)	Range (mg/L)	S ≥ cutoff (mg/L)	MIC_50_ (mg/L)	MIC_90_ (mg/L)	Susceptibility (%)
Ceftaroline	≤0.12–2	1–2*	≤0.12	1	90.5
Ceftobiprole	≤0.12– > 8	2*	1	8	76.2
Clindamycin	≤0.06– > 1	0.25	0.12	0.25	76.2
Levofloxacin	≤0.12– > 4	0.001–1	0.25	4	69.8
Delafloxacin	0.002–1	0.25*	0.008	0.5	74.6
Vancomycin	1–2	2	1	2	100.0
Teicoplanin	0.5–8	4	1	4	98.4
Daptomycin	≤0.06–4	1	1	2	87.3
Dalbavancin	≤0.03–0.25	0.125	0.06	0.12	98.4
Rifampicin	≤0.03– > 4	0.06	≤0.03	0.03	93.7
Linezolid	≤0.5– > 16	4	1	2	98.4
Tedizolid	0.12– > 2	0.5	0.25	0.25	98.4
Trimethoprim-sulfamethoxazole	≤0.25– > 4	2–4	≤0.25	0.5	98.4

Disc diffusion IZD_50/90_ (mm) and MIC_50/90_ (mg/L) values are reported together with susceptibility rates (%) by antibiotic. Breakpoints were interpreted according to EUCAST v15.0 for *Staphylococcus* spp., *S. aureus* (*) and *S. saprophyticus* (**) breakpoints (where applicable). For kanamycin and fosfomycin, EUCAST v13.1 (***) and CASFM 2020 v1.2 (****) breakpoints were used, respectively.

Regarding macrolides-lincosamides-streptogramins (MLS), 41 isolates (65.1%) were susceptible to erythromycin (IZD_50/90_ = 35/6 mm) and 48 isolates (76.2%) were susceptible to clindamycin (IZD_50/90_ = 35/6 mm, MIC_50/90_ = 0.12/0.25 mg/L). Based on susceptibility profiles and D-zone testing, 15 isolates (23.8%) exhibited a constitutive MLS_B_ phenotype, five (7.9%) an inducible MLS_B_ phenotype, and two (3.2%) an efflux-mediated resistance.

For fluoroquinolones, norfloxacin screening classified 46 isolates (73.0%) as susceptible; however, levofloxacin susceptibility was slightly lower at 69.8% (*n* = 44, MIC_50/90_ 0.25/4 mg/L). Three levofloxacin-resistant isolates (MICs 2-≥4 mg/L) were misclassified as susceptible by norfloxacin screening (zones 22–25 mm), raising concerns about potential false-negative results in clinical settings (Supplementary Figure 4). Delafloxacin susceptibility was 74.6% (*n* = 47), with MIC_50/90_ values of 0.008/0.5 mg/L.

For aminoglycosides, 60 isolates (95.2%) were susceptible to kanamycin, and 62 isolates (98.4%) were susceptible to both tobramycin and gentamicin (IZD_50/90_ = 32/28 mm, 36/32 mm, and 36/32 mm, respectively). One isolate (MSP009) was resistant to trimethoprim-sulfamethoxazole, whereas three isolates (RSP039, LSP2011, SP165) were classified as susceptible at increased exposure using disc diffusion. When tested by determining the MIC, resistance was confirmed only for MSP009, yielding trimethoprim-sulfamethoxazole susceptibility rates of 93.7% (disc diffusion) and 98.4% (MIC). IZD_50/90_ values were 32/22 mm, and MIC_50/90_ values were ≤0.25/0.5 mg/L. Tetracycline susceptibility was high (96.8%, *n* = 61). All isolates were susceptible to vancomycin (MIC_50/90_ = 1/2 mg/L). One isolate (RSP010) was resistant to teicoplanin, with MIC_50/90_ values (1/4 mg/L) close to the EUCAST resistance threshold (>4 mg/L). Daptomycin MIC_50/90_ values were elevated (1/2 mg/L), with eight resistant isolates, resulting in an overall susceptibility rate of 87.3%. All isolates remained susceptible to dalbavancin (MIC_50/90_ = 0.06/0.12 mg/L). High susceptibility was also observed for rifampicin (93.7%, *n* = 59) and for fusidic acid (92.1%, *n* = 58). One isolate (LSP0671) was resistant to oxazolidinones, whereas linezolid and tedizolid MIC_50/90_ values were 1/2 mg/L and 0.25/0.25 mg/L, respectively. In contrast, fosfomycin resistance was common (92.1%, *n* = 58), with uniformly small inhibition zones (IZD_50/90_ = 6/6 mm).

Overall, 22 isolates (35.4%) were multidrug-resistant (i.e. resistant to at least three antibiotic classes). For aminoglycosides, trimethoprim-sulfamethoxazole, rifampicin, fusidic acid, tetracycline, and quinupristin-dalfopristin, IZD_50_ and IZD_90_ values exceeded susceptibility cut-offs, indicating that ≥90% of isolates were susceptible. By contrast, penicillin G, cefoxitin, erythromycin, and clindamycin displayed IZD_90_ values of 6 mm, indicating low susceptibility rates. Using dilution methods, all MIC_50_ values were below resistance thresholds, except for daptomycin. MIC_90_ values met or exceeded breakpoints for antibiotics with ≤90% susceptibility, including ceftobiprole, clindamycin, levofloxacin, delafloxacin, and daptomycin.

**Figure 2.**
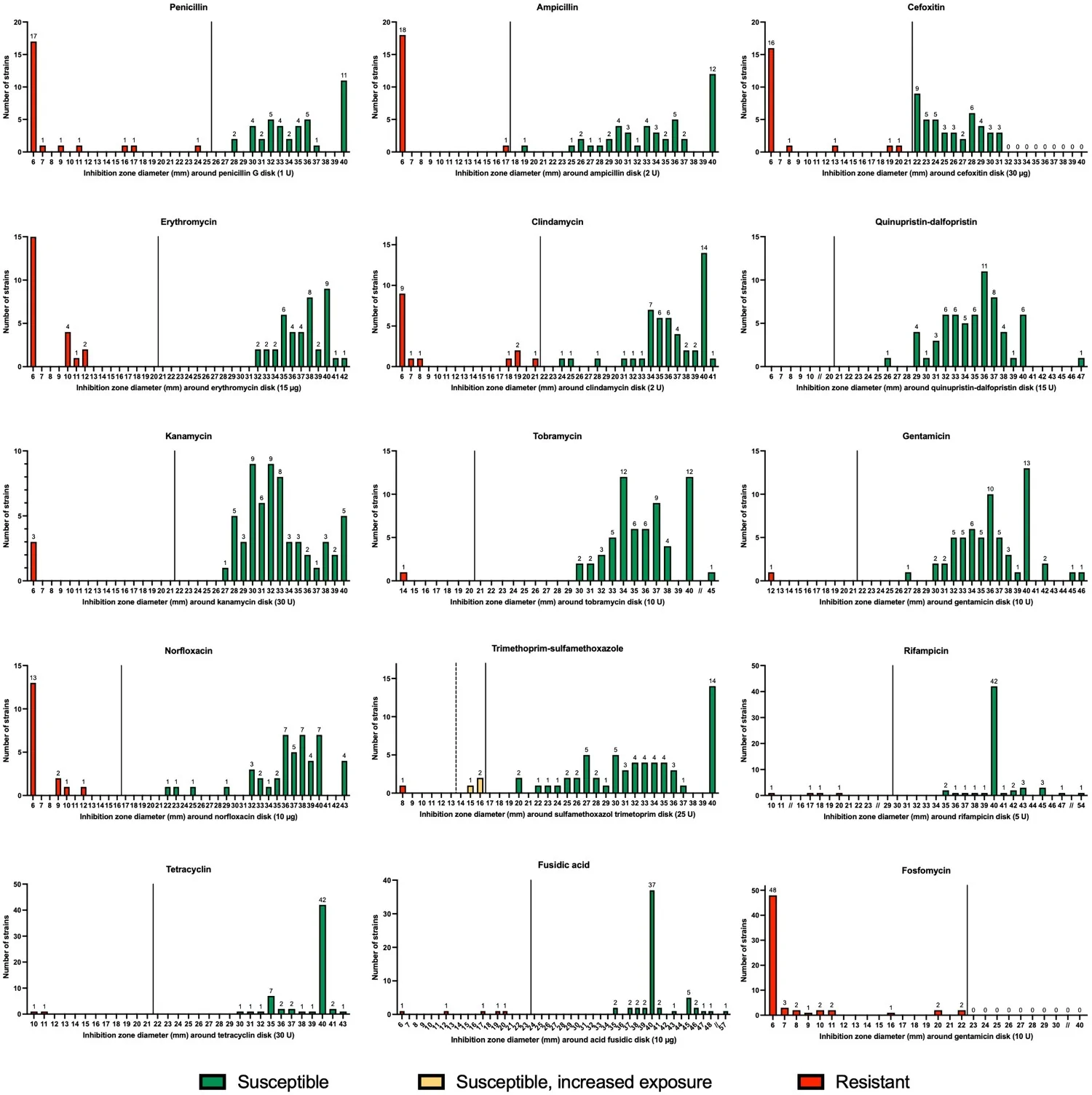
Distribution of inhibition zone diameters (IZDs) for the antibiotics tested by disk diffusion in the 61 *S. pettenkoferi* isolates, the type strain CCUG51270^T^, and the reference strain K6999 (*n* = 63). Histograms show the frequency distribution of IZDs (mm) for each antimicrobial agent across the collection. Colour scheme: Green bars represent susceptible (S) isolates, red bars resistant (R) isolates, and yellow bars isolates categorized as susceptible at increased exposure (I). Interpretation is based on EUCAST v15.0 breakpoints, with resistant cut-offs shown as solid black lines and susceptible at increased exposure (where applicable) as black dotted lines. For kanamycin and fosfomycin, EUCAST v13.1 and CASFM 2020 v1.2 diameter breakpoints were used, respectively.

**Figure 3.**
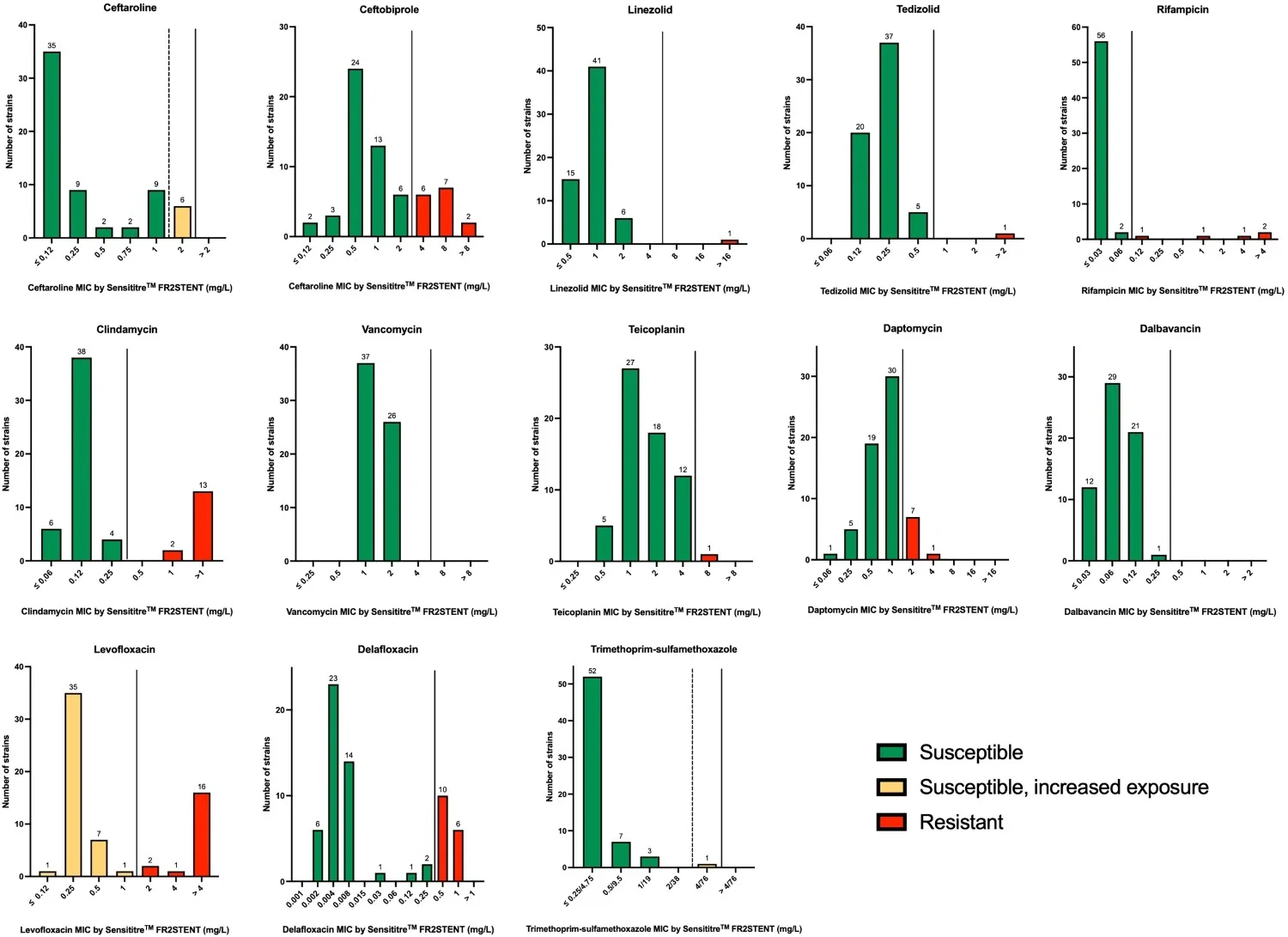
Distribution of Minimal Inhibitory Concentrations (MICs) for the antibiotics tested by broth microdilution method (Sensititre™) in the 61 *S. pettenkoferi* isolates, the type strain CCUG51270^T^, and the reference strain K6999 (*n* = 63). Histograms show the distribution of MIC values (mg/L) for each antimicrobial agent. Colour scheme: Green indicates susceptible (S) isolates, red resistant (R) isolates, and yellow isolates categorized as susceptible at increased exposure (I). Interpretation follows EUCAST v15.0 breakpoints, with R and I (where applicable) cut-offs are represented by solid and dotted black lines, respectively. For ceftaroline, ceftobiprole, dalbavancin, and delafloxacin, *S. aureus* breakpoints were applied.

Inhibition zone diameter distributions showed distinct susceptible and resistant populations for most antibiotics, except for ampicillin, cefoxitin, clindamycin, trimethoprim-sulfamethoxazole, and fosfomycin (Figure 2). MIC distributions showed that susceptible populations were generally separated from the EUCAST cut-offs by at least one dilution step. However, ceftobiprole, teicoplanin, and daptomycin exhibited MIC distributions very close to their respective breakpoints (Figure 3).

### Antimicrobial resistance genes and their correlation with phenotypic profiles

**Figure 4.**
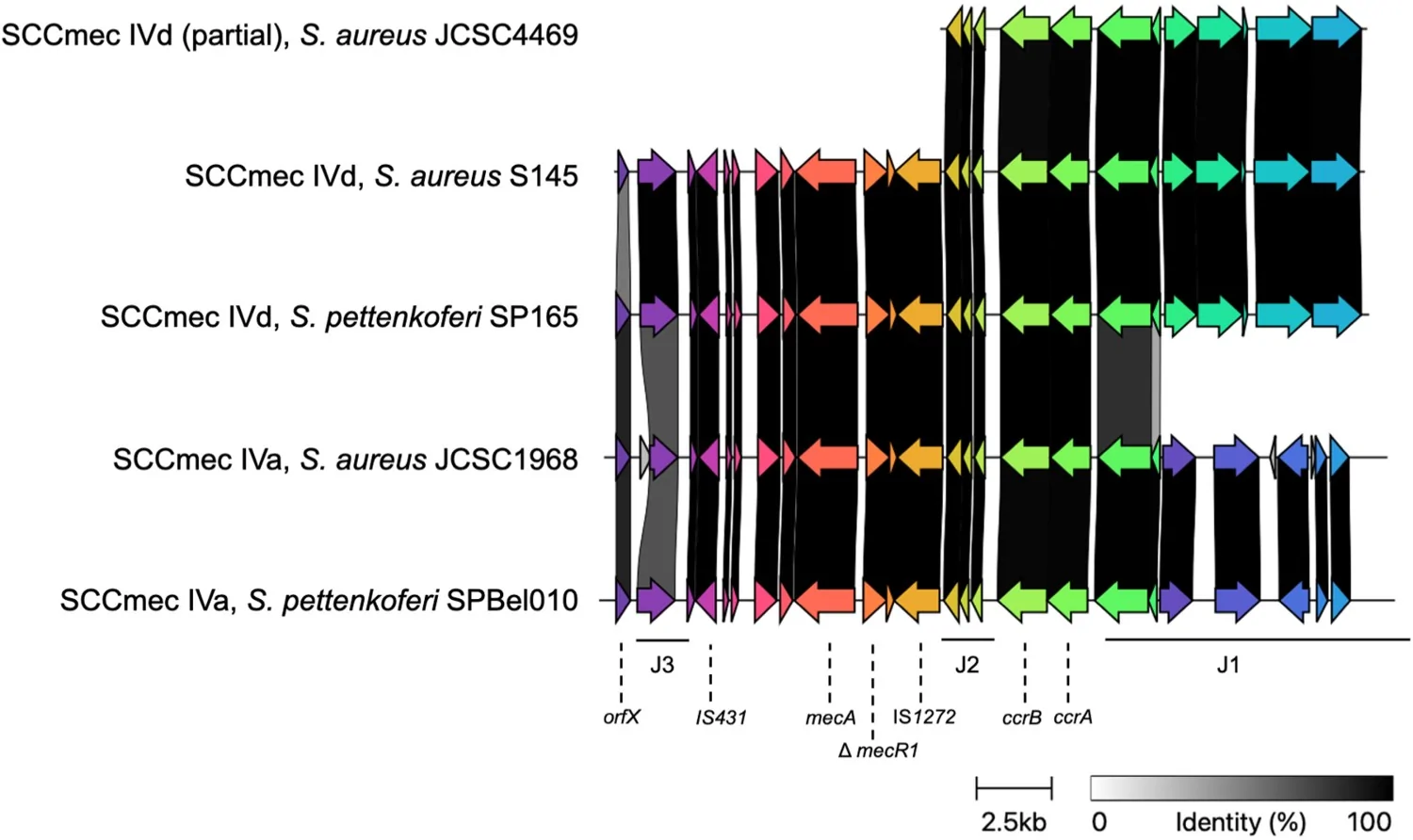
Comparative genomic organization of the SCC*mec* IVd(2B) and SCC*mec* IVa(2B) elements identified in *S. pettenkoferi*. Comparative gene organization of the representative SCC*mec* IVd(2B) element from isolate SP165 and the SCC*mec* IVa(2B) element from isolate SPBel010 with the corresponding reference SCC*mec* elements from *Staphylococcus aureus* JCSC4469 (partial SCC*mec* IVd(2B), GenBank accession AB097677.1), *S. aureus* S145 (complete SCC*mec* IVd(2B), BioSample SAMN24290962), and *S. aureus* JCSC1968 (SCC*mec* IVa, GenBank accession AB063172). SCC*mec* regions were independently annotated using Bakta and DFAST, and gene synteny was visualized using CAGECAT (clinker). Genes are coloured according to functional annotation, and shaded links indicate homologous proteins.

Among the 63 *S. pettenkoferi* isolates, 13 (20.6%) carried *blaZ* gene, and 18 (28.6%) carried *mecA* embedded within SCC*mec* elements. Seventeen isolates harboured SCC*mec* IVd(2B) (100% identity for core genes, >85% template coverage), whereas strain SPBel010 carried an SCC*mec* IVa(2B) cassette (100% identity for core genes, 88.2% template coverage). Comparative analysis of representative SCC*mec* elements showed conserved gene organization relative to the corresponding reference SCC*mec* IVd(2B) and IVa elements (Figure 4). While the *mec* and *ccr* complexes were conserved across all elements, the IVd and IVa subtypes mainly differed in the organization and gene content of the J1 region, with only limited variation observed in the J3 region. As most isolates were sequenced using short-read technology only, these SCCmecFinder subtype assignments should nonetheless be interpreted with some caution outside the two isolates (SP165 and SPBel010) for which comparative structural confirmation was obtained. Overall genotype-phenotype correlations are summarized in Table 3.

**Table 3.** Genotype-phenotype correlation of antimicrobial resistance determinants in *S. pettenkoferi*. Acquired resistance genes and chromosomal mutations are grouped according to antimicrobial class and their corresponding phenotypic resistance profiles. The table summarizes the frequency of each determinant, the associated resistance phenotype(s), and the degree of genotype-phenotype concordance.

Class	Resistance determinant (s)	Associated phenotype	Genotype-phenotype concordance
β-lactams	*blaZ,*13 (20.6)	Penicillin G (R) 23 (36.5) Ampicillin (R) 20 (31.7)	Partial 1 false susceptible; 2 false resistant isolates (Penicillin G) 3 false susceptible isolates(Ampicillin)
	*mecA* (SCCmec IVd, *n* = 17; SCCmec IVa, *n* = 1) 18 (28.6)	Cefoxitin (R) 20 (31.7) Ceftobiprole (R) 15 (23.8) Ceftaroline (I) 6 (9.5)	Partial 2 false resistant *mecA/mecC*-negative isolates All ceftobiprole-resistant isolates were *mecA*-positive
MLS_B_	*erm(A)*, *erm(C)* 20 (31.7)	cMLSB, 15 (23.8) iMLSB, 5 (7.9)	Complete
	*msr(A)* 2 (3.2)	MS phenotype (efflux-mediated), 2 (3.2)	
	*vga(A)* 26 (41.3)	Quinupristin-dalfopristin (R) 0 (0)	No association
Aminoglycosides	*ant(9)-Ia* 19 (30.2)	Spectinomycin (not tested)	Not evaluated
	*aph(3′)-III* 2 (3.2)	Kanamycin (R) 2 (3.2)	Complete
	*aac(6′)-aph(2″)* 1 (1.6)	Gentamicin/Tobramycin/Kanamycin (R) 1 (1.6)	Complete
Fusidic acid	*fusB* 4 (6.3)	Fusidic acid (R) 4 (6.3)	Complete
	*fusA* mutations (S65L and H467C) 1 (1.6)	Fusidic acid (R) 1 (1.6)	Complete
Tetracyclines	*tet(K)* 2 (3.2)	Tetracycline (R) 2 (3.2)	Complete
Rifamycins	*rpoB* mutations (D461Y or H481) 4 (6.3)	Rifampicin (R) 5 (7.9)	Partial 1 resistant isolate lacked detectable mutation
Fluoroquinolones	QRDR mutations in *gyrA* and *parC* 20 (31.7)	Fluoroquinolone (R) 20 (31.7)	Complete
Glycopeptides/Lipopeptides	*cls1* A215T	Elevated teicoplanin and daptomycin MICs	Strong association
	*walK*/*walR* variants		Frequently co-occurring with *cls1* A215T
	*cls2* + *walR* variants		Putative alternative pathway (LSP1043)
	*mprF* substitutions	Variable phenotype	No association
Antifolates	*folA* L21 V + *folP* P112R 6 (9.5)	Trimethoprim-sulfamethoxazole (I) 3 (4.8)	Partial Also detected in two susceptible isolates
	*dfrC* 1 (1.6)	Trimethoprim-sulfamethoxazole (R) 1 (1.6)	Complete
Oxazolidinones	*rrl* G2576 T + *rplC* M156T 1 (1.6)	Linezolid/Tedizolid (R) 1 (1.6)	Complete
Fosfomycin	*fosB*-like variant A 58 (92.1)	Fosfomycin (R) 58 (92.1)	Partial Testing limitations
	*fosB*-like variant B 4 (6.3)	Fosfomycin (S) 4 (6.3)	
Other resistance determinants	*mupA,* 2 (3.2)	Predicted high-level mupirocin resistance (not phenotypically tested)	Not evaluated
	*qac,* 1 (1.6)	Predicted reduced susceptibility to quaternary ammonium compounds (not phenotypically tested)	Not evaluated

Abbreviations: R, resistant; I, susceptible, increased exposure; cMLSB, constitutive macrolide-lincosamide-streptogramin B phenotype; iMLSB, inducible macrolide-lincosamide-streptogramin B phenotype; MS, macrolide-streptogramin phenotype; QRDR, quinolone resistance-determining region; MIC, minimum inhibitory concentration. *Complete* indicates complete agreement between genotype and phenotype. *Partial* indicates that the resistance determinant was associated with the corresponding phenotype but genotype-phenotype discordance was observed in one or more isolates. *Strong association* indicates mutations consistently associated with the corresponding phenotype without complete one-to-one concordance. *No association* indicates the absence of a consistent genotype-phenotype relationship. *Not evaluated* indicates that the corresponding phenotype was not tested. Cell shading denotes the genotype–phenotype concordance category: green indicates *Complete* concordance, orange indicates *Partial* concordance or *Strong association*, red indicates *No association*, and grey indicates *Not evaluated*. Variants A and B represent the two *fosB*-like proteins identified in this study (annotated as WP_000920239.1; 68.1% amino acid identity, 99.3% coverage, E-value 6.0 × 10^−^⁵⁴; and WP_002437790.1; 65.7% amino acid identity, 98.6% coverage, E-value 1.3 × 10^−^⁵³, respectively).

**Figure 5.**
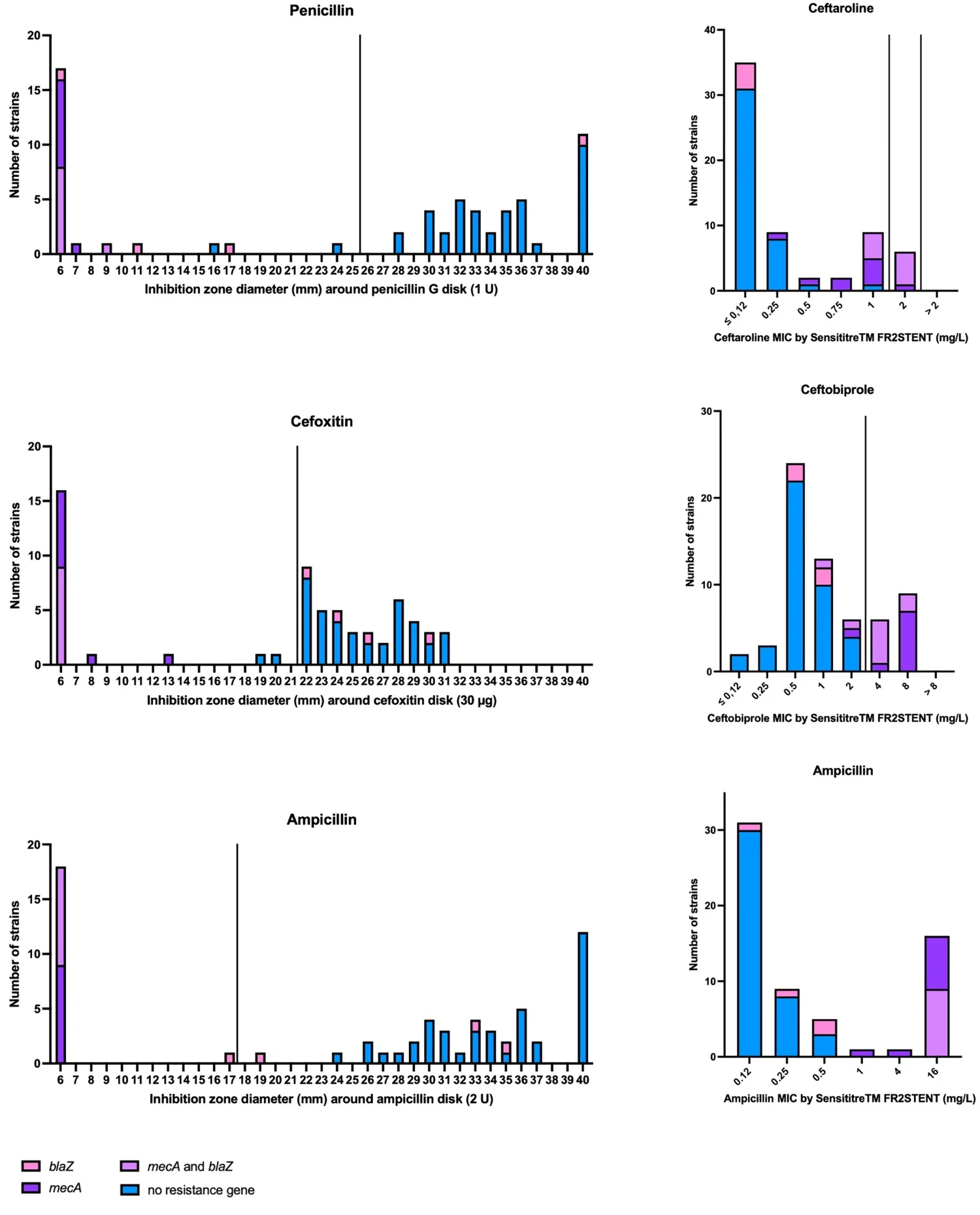
Correlation between genotype and phenotype for beta-lactam susceptibility in *S. pettenkoferi*. Scatter or violin plots (as appropriate) show the relationship between IZDs (mm) or MICs (mg/L) and the presence/absence of *mecA* and *blaZ*. EUCAST v15.0 breakpoints are indicated by solid (R) and dotted (I) lines. Cefoxitin screening classification, ceftobiprole-R isolates, and ceftaroline-I isolates are highlighted to illustrate concordance and misclassification patterns.

Among β-lactam resistance determinants, *mecA* (encoding PBP2a) conferred methicillin resistance and was present in all ceftobiprole-resistant isolates, including the six isolates requiring increased exposure to ceftaroline (Figure 5). Cefoxitin correctly identified all *mecA*-positive isolates but misclassified two *mecA/mecC*-negative isolates as resistant. In contrast, penicillin G and ampicillin disks showed limited performance for *blaZ* detection, resulting in both false-susceptible and false-resistant classifications (Figure 5 and Table 3).

Resistance to MLS compounds was explained by the presence of *erm(A)*, *erm(C)* and *msr(A)* genes. All 22 erythromycin-resistant isolates carried *erm(A)* (*n* = 19), *msr(A)* (*n* = 2; MSP019 and RSP014), or *erm(C)* (*n* = 1; MSP009). Among these, the 15 clindamycin-resistant isolates exhibited a constitutive MLS_B_ (cMLS_B_) phenotype, the five *erm(A)*-positive, clindamycin-susceptible isolates exhibited an inducible MLS_B_ (iMLS_B_) phenotype, and the two *msr(A)*-positive isolates displayed the M phenotype. In contrast, *vga(A)*, despite being detected in 26 isolates (41.3%), was not associated with phenotypic resistance to quinupristin-dalfopristin (Table 3).

Aminoglycoside resistance determinants showed complete genotype-phenotype concordance (Table 3). The *ant(9)-Ia* gene, associated with spectinomycin resistance (not phenotypically tested), was detected in 19 isolates, including all 18 MRSP isolates (Figure 1). Strain MSP009 uniquely carried *aac(6′)-aph(2″)*, explaining high-level resistance to kanamycin, tobramycin, and gentamicin. For fusidic acid, *fusB* was detected in four of the five resistant isolates, whereas strain 12-586776 carried *fusA* substitutions (S65L and H467C), suggesting an alternative resistance mechanism. Four rifampicin-resistant isolates carried *rpoB* mutations (H481N in SP165 and RSP006; D461Y in LSP2199 and NSP031P), whereas one isolate (RSP014), resistant only by MIC testing, lacked detectable *rpoB* mutations (Table 3).

Fluoroquinolone resistance showed complete concordance with mutations in the quinolone resistance-determining regions (QRDRs) of *gyrA* and *parC* (Table 3). The predominant *gyrA* substitutions were S84L (*n* = 17) and S84A (*n* = 3), with E88 K additionally detected in two isolates. All resistant isolates carried concomitant *parC* substitutions (T80 K/I/R, S81P, or D84G/Y/N), whereas among susceptible isolates only RSP006 harboured a single *parC* T80M substitution without phenotypic impact. Sixteen isolates were resistant to all three tested fluoroquinolones (norfloxacin, levofloxacin, and delafloxacin); three isolates (NSP009P, NASP011, and SPBel010) were resistant only to levofloxacin; and one isolate (505016231486) was resistant only to norfloxacin.

For glycopeptides and lipopeptides, multiple substitutions were identified in *mprF* (A198 V, M221I, S370F, S424 T, L526 V, D747N, and M820I), although none were consistently associated with teicoplanin or daptomycin resistance. By contrast, *cls1* A215 T was consistently associated to elevated MICs for both teicoplanin and daptomycin. These isolates also harboured recurrent mutations in *walK* (e.g. D66E, I218F, E302D, E509D, R263C, and G508 V), and less frequently, in *walR* (Q34E, E39D, A40 T, A45S, L91I, E323Q, S338 T, G339E, D351G, D373E, and V407I). The divergent isolate (LSP1043) carried multiple *cls2* substitutions (D130Y, H132Q, M243 T, V246I, N313D, A340E) combined with *walR* variants (V119M, T336M), suggesting an alternative pathway towards reduced susceptibility.

For trimethoprim-sulfamethoxazole, three isolates categorized as susceptible at increased exposure carried combined *folA*(L21 V) and *folP* (P112R) mutations. This pattern was absent from fully susceptible isolates, except for MSP004 and NSP031P, which exhibited borderline inhibition zone diameters. Strain MSP009 carried *dfrC*, explaining high-level resistance.

The linezolid-resistant isolate LSP0671 carried *rplC* M156 T and *rrl* G2576 T, the latter previously associated with linezolid resistance in *S. epidermidis* [27]. No *rlpD* mutations were detected. LSP0671 also exhibited resistance to methicillin, ceftobiprole, erythromycin, clindamycin, fluoroquinolones, tedizolid, and fosfomycin.

Nearly all isolates carried a *fosB*-like gene, except LSP1043. Two distinct *fosB*-like variants were identified, one predominating among the 58 fosfomycin-resistant isolates and the other among the four isolates categorized as susceptible by disk diffusion (MSP005, MSP016, NASP009, and RSP010) (Table 3). This apparent genotype-phenotype discordance likely reflects the recognized limitations of fosfomycin susceptibility testing for this species [23]. Two isolates (RSP039, LSP2011) carried *mupA* gene, conferring high-level mupirocin resistance, and one (LSP2199) carried *qac*, associated with reduced susceptibility to quaternary ammonium compounds.

### Distribution of antimicrobial resistance across clades, geographical origins, infection sites, and DFIs

Statistical analyses were performed to assess associations between phylogenetic clades and antimicrobial resistance profiles (Table 4). Strain LSP1043, identified as an outlier, was excluded from all clade-related analyses.

**Table 4.** Phenotypic antimicrobial resistance across *S. pettenkoferi* clades.

Antibiotics	All isolates (*n* = 63)	Clade				*p*-value	*q*-value	Clade				*p*-value	*q*-value
		I-A (*n* = 47)	I-B (*n* = 4)	II-A (*n* = 8)	II-B (*n* = 3)			I-A (*n* = 47)	I-B (*n* = 4)	II-A (*n* = 8)	II-B (*n* = 3)		
		Phenotypical resistance N (%)						IZD (mm) or MIC (mg/L) Median [IQR]					
PEN	23 (37)	21 (45)	1 (25)	1 (13)	0 (0)	0.2	0.5	30 [6, 36]	31 [21, 34]	35 [32, 39]	33 [31, 35]	0.2	0.4
AMP	20 (32)	18 (38)	1 (25)	1 (13)	0 (0)	0.4	0.7	30 [6, 35]	39 [28, 40]	33 [29, 38]	36 [33, 36]	0.08	0.2
FOX	20 (32)	20 (43)	0 (0)	0 (0)	0 (0)	0.02*	0.5	22 [6, 27]	25 [23, 27]	24 [23, 27]	26 [24, 28]	0.3	0.4
CPT	0 (0)	0 (0)	0 (0)	0 (0)	0 (0)	–	–	0.25 [0.12, 1.00]	0.12 [0.12, 0.19]	0.12 [0.12, 0.19]	0.12 [0.12, 0.12]	0.08	0.2
BPR	6 (9.5)	15 (32)	0 (0)	0 (0)	0 (0)	0.13	0.5	1.00 [0.50, 4.00]	0.50 [0.38, 0.50]	0.75 [0.50, 1.00]	0.25 [0.12, 0.50]	0.003**	0.086
ERY	22 (35)	20 (43)	0 (0)	2 (25)	0 (0)	0.2	0.5	35 [6, 38]	35 [34, 38]	36 [19, 40]	38 [35, 38]	0.7	0.8
CLI	15 (24)	13 (28)	0 (0)	2 (25)	0 (0)	0.7	>0.9	0.12 [0.12, 1.00]	0.12 [0.12, 0.12]	0.12 [0.09, 0.63]	0.12 [0.06, 0.12]	0.3	0.5
QDA	0 (0)	0 (0)	0 (0)	0 (0)	0 (0)	–	–	36.0 [33.0, 37.0]	31.0 [29.0, 36.5]	34.0 [33.0, 36.0]	35.0 [31.0, 36.0]	0.5	0.6
NOR	17 (27)	17 (36)	0 (0)	0 (0)	0 (0)	0.06	0.5	35 [6, 39]	36 [34, 37]	37 [30, 38]	38 [36, 40]	0.6	0.7
LVX	19 (30)	17 (36)	0 (0)	2 (25)	0 (0)	0.4	0.7	0.25 [0.25, 4.00]	0.25 [0.25, 0.38]	0.38 [0.25, 1.25]	0.25 [0.25, 0.25]	0.3	0.5
DLX	16 (25)	16 (34)	0 (0)	0 (0)	0 (0)	0.09	0.5	0.008 [0.004, 0.50]	0.003 [0.002, 0.004]	0.008 [0.008, 0.019]	0.004 [0.002, 0.004]	0.015*	0.11
KAN	3 (4.8)	2 (4.3)	0 (0)	1 (13)	0 (0)	0.6	0.9	33.0 [30.0, 36.0]	28.0 [28.0, 29.0]	32.0 [30.0, 34.5]	30.0 [27.0, 32.0]	0.03*	0.14
TOB	1 (1.6)	0 (0)	0 (0)	1 (13)	0 (0)	0.2	0.5	36.00 [34.00, 38.00]	32.50 [31.00, 34.50]	34.00 [32.00, 39.00]	33.00 [31.00, 35.00]	0.03*	0.14
GEN	1 (1.6)	0 (0)	0 (0)	1 (13)	0 (0)	0.2	0.5	36.0 [34.0, 40.0]	31.0 [28.5, 36.0]	34.5 [32.5, 38.0]	33.0 [32.0, 36.0]	0.08	0.2
VAN	0 (0)	0 (0)	0 (0)	0 (0)	0 (0)	–	–	1.00 [1.00, 2.00]	2.00 [2.00, 2.00]	1.00 [1.00, 1.50]	2.00 [2.00, 2.00]	0.008**	0.11
TEC	1 (1.6)	0 (0)	1 (25)	0 (0)	0 (0)	0.11	0.5	1.00 [1.00, 2.00]	4.00 [4.00, 6.00]	1.00 [1.00, 2.00]	2.00 [1.00, 2.00]	0.02*	0.11
DAP	8 (13)	5 (11)	1 (25)	0 (0)	1 (33)	0.2	0.5	1.00 [0.50, 1.00]	1.00 [1.00, 1.50]	0.50 [0.50, 0.75]	1.00 [1.00, 2.00]	0.03*	0.14
DAL	1 (1.6)	1 (2.1)	0 (0)	0 (0)	0 (0)	>0.9	>0.9	0.060 [0.060, 0.120]	0.060 [0.045, 0.090]	0.120 [0.060, 0.120]	0.060 [0.060, 0.060]	0.4	0.6
TET	3 (3.1)	1 (2.1)	0 (0)	1 (13)	0 (0)	0.4	0.7	40.00 [38.00, 40.00]	36.00 [35.00, 38.50]	40.00 [38.00, 40.00]	40.00 [40.00, 40.00]	0.3	0.4
FUS	5 (7.9)	4 (8.5)	0 (0)	0 (0)	0 (0)	>0.9	>0.9	40 [40, 40]	40 [37, 40]	40 [40, 43]	40 [37, 40]	0.2	0.4
LZD	1 (1.6)	1 (2.1)	0 (0)	0 (0)	0 (0)	>0.9	>0.9	1.00 [1.00, 1.00]	0.75 [0.50, 1.00]	1.00 [1.00, 1.00]	1.00 [0.50, 1.00]	0.3	0.4
TZD	1 (1.6)	1 (2.1)	0 (0)	0 (0)	0 (0)	>0.9	>0.9	0.25 [0.12, 0.25]	0.12 [0.12, 0.19]	0.25 [0.19, 0.44]	0.25 [0.12, 0.25]	0.2	0.4
RIF	5 (7.9)	4 (8.5)	0 (0)	0 (0)	0 (0)	>0.9	>0.9	0.03 [0.03, 0.03]	0.03 [0.03, 0.03]	0.03 [0.03, 0.03]	0.03 [0.03, 0.03]	0.5	0.6
SXT	1 (1.6)	0 (0)	0 (0)	1 (13)	0 (0)	0.2	0.5	0.25 [0.25, 0.25]	0.25 [0.25, 0.25]	0.25 [0.25, 0.25]	0.25 [0.25, 0.50]	0.7	0.8
FOS	58 (92.1)	47 (100)	0 (0)	8 (100)	3 (100)	0.16	0.5	6.0 [6.0, 6.0]	29 [27.5, 31.5]	6.0 [6.0, 6.5]	6.0 [6.0, 6.0]	0.0001**	0.025

For each antibiotic, resistant isolates are shown as N (%), and IZD (mm) or MICs (mg/L) values are expressed as Median [IQR], stratified by clade/subclade. Statistical significance: *p < 0.05; **p < 0.01; ***p < 0.001. The outlier isolate LSP1043 was excluded.

Subclade I-A demonstrated higher cefoxitin resistance and higher ceftobiprole MICs than the other subclades. This subclade also exhibited increased delafloxacin resistance and elevated delafloxacin MICs. Resistance to erythromycin, clindamycin, norfloxacin, and levofloxacin was also more common in subclade I-A. Conversely, subclades I-B and II-B exhibited higher vancomycin and teicoplanin MICs, whereas subclade II-A showed lower daptomycin MICs. Finally, subclade I-B displayed smaller inhibition zone diameters for kanamycin and tobramycin, and larger inhibition zone diameters for fosfomycin, compared with the other subclades (Table 5). However, none of these associations remained statistically significant after Benjamini-Hochberg correction for multiple testing (all *q*-values > 0.05).

Regarding the distribution of resistance determinants across clades, all *mecA*-positive isolates were located within subclade I-A (Figure 1). Similarly, *gyrA* QRDR mutations were restricted to subclade I-A. Although *ermA* and *ant(9)-Ia* were also more frequent in subclade I-A, they were detected in other phylogenetic backgrounds. One noteworthy isolate, NSP009P (subclade II-A), carried *ermA* and *ant(9)-Ia* but lacked *mecA* (Figure 1), suggesting that these resistance determinants may disseminate independently of the methicillin-resistant lineage. However, none of the observed associations between resistance determinants and phylogenetic subclades remained statistically significant after Benjamini-Hochberg correction for multiple testing (Table 5; all *q*-values > 0.05).

**Table 5.** Distribution of antimicrobial resistance determinants across *S. pettenkoferi* clades.

	Resistance genes	All isolates (n = 63)	Clade				*p*-value	*q*-value
			I-A (*n* = 47)	I-B (*n* = 4)	II-A (*n* = 8)	II-B (*n* = 3)		
			Phenotypical resistance N (%)					
Penicillin	*blaZ*	13 (21)	11 (23)	1 (25)	1 (13)	0 (0)	>0.9	>0.9
Methicillin	*mecA*	18 (29)	18 (38)	0 (0)	0 (0)	0 (0)	0.049*	0.4
Macrolide Lincosamide Streptogramin	*erm*(A)	19 (30)	18 (38)	0 (0)	1 (13)	0 (0)	0.2	0.5
	*erm*(C)	1 (1.6)	0 (0)	0 (0)	1 (13)	0 (0)	0.2	0.5
	*msr*(A)	2 (3.2)	2 (4.3)	0 (0)	0 (0)	0 (0)	>0.9	>0.9
	*vga*(A)	26 (41)	19 (40)	4 (100)	1 (13)	2 (67)	0.016*	0.2
Aminoglycosides	*aph(3')-III*	2 (3.2)	2 (4.3)	0 (0)	0 (0)	0 (0)	>0.9	>0.9
	*aac(6’)-aph(2’’)*	1 (1.6)	0 (0)	0 (0)	1 (13)	0 (0)	0.2	0.5
	*ant(9)-Ia*	19 (30)	18 (38)	0 (0)	1 (13)	0 (0)	0.2	0.5
Tetracycline	*tet*(K)	2 (3.2)	1 (2.1)	0 (0)	1 (13)	0 (0)	0.4	0.8
Quinolone	*gyrA*	18 (29)	18 (38)	0 (0)	0 (0)	0 (0)	0.049*	0.3
	*parC*	21 (33.3)	19 (40.4)	0 (0)	2 (25)	0 (0)	0.3	0.5
Trimethoprim-sulfamethoxazole	*dfrC*	1 (1.6)	0 (0)	0 (0)	1 (13)	0 (0)	0.2	0.5
	*folA* L21 V associated with *folP* P112R	6 (9.5)	6 (12.8)	0 (0)	0 (0)	0 (0)	0.80	>0.9
Oxazolidinone	*rrl* G2576T	1 (1.6)	1 (2.1)	0 (0)	0 (0)	0 (0)	>0.9	>0.9
Fusidic acid	*fusB*	3 (4.8)	3 (6.4)	0 (0)	0 (0)	0 (0)	>0.9	>0.9
	*fusA* S65L and H467C	1 (1.6)	1 (2.1)	0 (0)	0 (0)	0 (0)	>0.9	>0.9
Fosfomycin	*fosB*	62 (98.4)	47 (100)	4 (100)	8 (100)	3 (100)	–	–
Rifampicin	*rpoB* H481N	2 (3.2)	2 (4.3)	0 (0)	0 (0)	0 (0)	>0.9	>0.9
	*rpoB* D471Y	2 (3.2)	2 (4.3)	0 (0)	0 (0)	0 (0)	>0.9	>0.9
Mupirocin	*mupA*	2 (3.2)	2 (4.3)	0 (0)	0 (0)	0 (0)	>0.9	>0.9
Antiseptic	*qacG*	1 (1.6)	1 (2.1)	0 (0)	0 (0)	0 (0)	>0.9	>0.9

For each antibiotic class, the presence of acquired resistance genes and key mutations is reported as N (%) per clade/subclade. Statistical significance is indicated as follows: *p-value < 0.05; **p-value < 0.01; ***p-value < 0.001.

Potential associations between resistance profiles and geographical origin, clinical sample type, and infection context (DFIs vs. non-DFI) were evaluated (Supplementary Tables 6–11). Isolates were categorized into four groups: France, Germany, Sweden, and Belgium, with the single isolate from Switzerland excluded from this analysis. No significant differences were observed in either phenotypic or genotypic resistance across countries (Supplementary Tables 6 and 7). However, several trends were noted. German isolates showed higher vancomycin MICs (*p* = 0.003); Swedish isolates exhibited lower daptomycin MICs (0.5 mg/L vs. 1 mg/L for other countries, *p* = 0.02); and French isolates displayed smaller trimethoprim-sulfamethoxazole IZDs (*p* = 0.04).

Regarding clinical sample type, rifampicin resistance was significantly associated with BJIs and non-bloodstream samples (*p* = 0.006). Importantly, isolates from DFIs or DFOMs exhibited higher rifampicin resistance (20% vs. 2.1%, *p* = 0.039) and larger IZDs for kanamycin and tobramycin (*p* = 0.037 and *p* = 0.039, respectively).

These findings collectively highlight a consistent clustering of resistance traits within subclade I-A, alongside subtle geographical and clinical influences on antimicrobial susceptibility.

## Discussion

This study provides the first large-scale European investigation of *S. pettenkoferi,* an emerging CoNS species whose epidemiology, antimicrobial susceptibility, and genomic characteristics have remained insufficiently explored. By analysing 63 isolates collected over more than a decade and across multiple European countries, we elucidated the population structure of this species and identified antimicrobial resistance patterns with potential clinical implications.

Whole-genome phylogenetic reconstruction revealed a structured population composed of two major clades and four subclades, supported by high bootstrap values (e.g. 100), indicating robust genetic relatedness within *S. pettenkoferi* species. Subclade I-A emerged as the dominant lineage, spanning more than two decades and multiple European countries. Its inclusion of the type strain CCUG51270^T^and the reference strain K6999 and its enrichment in pre-2020 isolates suggest that this lineage may represent a stable, ancestral, and widely disseminated lineage within *S. pettenkoferi*. By contrast, subclades I-B, II-A, and II-B exhibited more restricted distribution patterns, which could reflect niche adaptation, restricted transmission, or more recent evolution. These smaller clusters warrant close genomic surveillance to track early signals of emerging resistance determinants. A notable feature was the presence of 18 highly related MRSP isolates within subclade I-A, with pairwise SNP distances ranging from 53 to 401 despite their broad geographic distribution. Their shared resistance determinants (SCC*mec* type IV, *erm(A), ant(9)-Ia*, mutations in *gyrA* and *parC*) suggest either clonal dissemination of a successful lineage or strong convergent evolution under similar antimicrobial selection pressures. This pattern mirrors findings in *S. epidermidis*, where SCC*mec* type IV lineages dominate hospital-associated CoNS populations [28], underscoring the need for coordinated international long-term genomic surveillance.

Among the 63 isolates, approximately one-third were multidrug-resistant, and 18 (28.6%) carried *mecA*, with SCC*mec* IVd(2B) (*mecA*, Δ*mecR1, IS*1272, *ccrA2*, and *ccrB2*) representing the predominant subtype (17/18; 94.4%). This aligns with observations in other CoNS species, where SCC*mec* type IV variants are among the most prevalent SCC*mec* elements and are thought to contribute substantially to the dissemination of methicillin resistance because of their relatively small size and genetic mobility [29–31]*.* The identification of a single SCC*mec* IVa(2B) element in SPBel010 emphasizes ongoing cassette mobility and the potential for horizontal gene transfer.

Although cefoxitin screening reliably detected *mecA*-positive isolates, it lacked full specificity: two Swedish isolates (13-289:1 and 505015575078) were falsely classified as methicillin-resistant (inhibition zones of 19 and 20 mm, respectively), despite lacking *mec* genes, and nine isolates had borderline cefoxitin IZDs without detectable resistance determinants. With a positive predictive value (PPV) of 90% in this dataset, cefoxitin screening alone may lead to unnecessary exclusion of beta-lactams in clinical practice. Specificity issues with cefoxitin testing have previously been reported for certain CoNS, particularly *S. epidermidis* and *S. capitis*, when evaluated using automated systems such as Vitek 2, further supporting the need for cautious interpretation of isolated cefoxitin results [32]. Similarly, penicillin G and ampicillin disks showed poor performance for *blaZ* detection, consistent with established limitations of beta-lactamase detection in other *Staphylococcus* species (except *S. aureus* and *S. saprophyticus*). These findings highlight the need to refine susceptibility testing rules for *S. pettenkoferi*. Fifth-generation cephalosporins exhibited heterogeneous activity against *S. pettenkoferi*. While ceftaroline remained largely effective, six MRSP isolates required increased exposure. By contrast, ceftobiprole resistance was relatively frequent (15 isolates), with MIC_50/90_ values (1/8 mg/L) substantially higher than those previously reported for other CoNS [33,34] and MRSA SCC*mec* type IV lineages [35,36]. This reduced susceptibility to ceftobiprole may represent a clinically relevant concern, particularly given its role as a last-line agent for multidrug-resistant Gram-positive infections. Glycopeptide susceptibility remained well preserved, with universal susceptible to vancomycin and only one teicoplanin-resistant isolate (RSP010). Dalbavancin also demonstrated excellent in vitro activity (MIC_50/90_ = 0.06-0.12 mg/L), consistent with previous findings [37]. In contrast, daptomycin showed elevated MICs (MIC_50/90_ = 1/2 mg/L), with eight resistant isolates, substantially exceeding the expected resistance rate of 0.1–0.3% reported for CoNS [38]. While daptomycin resistance is often associated with mutations affecting membrane homeostasis [39,40], our resistant isolates carried multiple combinations of mutations in *mprF, cls1*/*cls2*, and *walKR*, differing from previously published *S. pettenkoferi* resistance patterns [41–43]. This supports the idea that daptomycin resistance is multifactorial and species-specific, and highlights the need for functional validation.

Fluoroquinolone resistance was widespread, particularly for levofloxacin, with a susceptibility of <70%. Notably, norfloxacin disc screening underestimated resistance, misclassifying three levofloxacin-resistant isolates as susceptible. Given the clinical relevance of fluoroquinolones, especially in oral regimens for BJIs, development of more reliable screening criteria for *S. pettenkoferi* is warranted. As expected, fluoroquinolone resistance correlated strongly with canonical quinolone resistance-determining region mutations in *gyrA* and *parC*, as observed in other staphylococci, confirming conserved mechanisms across species.

Nearly all isolates carried a *fosB*-like gene, supporting a species-associated mechanism potentially contributing to intrinsic fosfomycin resistance, as described in *S. capitis* and *S. saprophyticus*. Discordances between genotype and phenotype were observed, in line with known limitations of fosfomycin susceptibility testing by disk diffusion or MIC for staphylococci. However, as fosfomycin is seldom used for BJIs, the clinical impact of this observation is limited. Other resistance determinants, including *tet(K)*, *fusB* and *dfrC*, as well as mutations in *fusA* and *rpoB*, were detected at low frequencies but showed complete concordance between genotype and phenotype.

This study has several limitations. Because isolates were collected retrospectively from multiple European centers, detailed and standardized clinical metadata were not consistently available. Consequently, associations between phylogenetic lineages, antimicrobial resistance determinants, and clinical outcomes could not be reliably assessed. In addition, the marked imbalance in the number of isolates across the phylogenetic subclades, particularly for subclades I-B and II-B, limited the statistical power of subgroup comparisons. Although multiple testing correction was applied, these analyses should therefore be considered exploratory and interpreted with caution. Future prospective studies integrating clinical and genomic data and including a more balanced representation of phylogenetic lineages are needed to better define the clinical significance of the genomic diversity observed in *S. pettenkoferi*. Furthermore, because the collection reflects a convenience sample contributed by participating centers rather than a systematic, prospective surveillance programme, the overall proportion of *S. pettenkoferi* among clinical isolates and the representativeness of the collection could not be determined.

Finally, this study shows the influence of phylogeny on antimicrobial susceptibility patterns, with subclade I-A being enriched in *mecA*, fluoroquinolone resistance mutations, and higher ceftobiprole and delafloxacin MICs. Subclades I-B and II-B exhibited higher glycopeptide MICs, whereas II-A displayed lower daptomycin MICs. Although geographical differences were not statistically significant overall, trends included higher vancomycin MICs among German isolates, lower daptomycin MICs in Swedish isolates, and higher rifampicin resistance among isolates from DFIs. Together, these findings illustrate the potential interplay between lineage, geography, and clinical context in shaping resistance patterns in *S. pettenkoferi*.

In conclusion, this study provides the most comprehensive genomic and phenotypic characterization of *S. pettenkoferi* to date, revealing the emergence of multidrug-resistant lineages, including isolates with reduced susceptibility to ceftobiprole and daptomycin. The detection of highly resistant, genetically cohesive lineages, particularly within subclade I-A, underscores the need for continued genomic surveillance, methodological refinement for susceptibility testing, and vigilant antimicrobial stewardship. These findings establish essential baseline data that may help inform future therapeutic strategies and support active epidemiological monitoring across Europe and beyond.

## Supplementary Material

SUPPLEMENTARY FIGURE.docx

Supplementary_Tables_030826.xlsx

## Data Availability

Whole-genome sequencing data generated in this study have been deposited in the NCBI GenBank database under the following BioProject accession numbers: PRJNA768314 (SP165), PRJNA870410 (NSP003H, NSP005H, NSP008P, NSP009P and NSP019P), and PRJNA1418137 (remaining isolates).
