# Supplementary material for: Staphylococcus pettenkoferi: an underrecognized species with clinically relevant antimicrobial resistance. A comprehensive genomic European study
Source: Emerg Microbes Infect. 2026 Sep 16;15(1):2724635. doi: 10.1080/22221751.2026.2724635 (PMC13587547; doi:10.1080/22221751.2026.2724635)
Supplement: Supplementary file 1 [file TEMI_A_2724635_SM2926.docx]

# SUPPLEMENTARY FIGURE

**Supplementary Figure 1.**

**Supplementary Figure 2.**

**Supplementary Figure 3.**

**Supplementary Figure 4.**
